# Hyperhomocysteinemia causes ER stress and impaired autophagy that is reversed by Vitamin B supplementation

**DOI:** 10.1038/cddis.2016.374

**Published:** 2016-12-08

**Authors:** Madhulika Tripathi, Cheng Wu Zhang, Brijesh Kumar Singh, Rohit Anthony Sinha, Kyaw Thu Moe, Deidre Anne DeSilva, Paul Michael Yen

**Affiliations:** 1 Laboratory of Hormonal Regulation, Cardiovascular and Metabolic Disorders Program, Duke-NUS Medical School, Singapore 169857, Singapore; 2 Stroke Trial Unit, Department of Neurology, Singapore General Hospital, Outram Road, Singapore 169608, Singapore; 3 National Neuroscience Institute, 11 Jalan Tan Tock Seng, Singapore 308433, Singapore; 4 Newcastle University Medicine Malaysia (NUMed, Malaysia) No. 1 Jalan Sarjana, Iskandar Puteri (formerly Nusajaya), Johor 179200, Malaysia

## Abstract

Hyperhomocysteinemia (HHcy) is a well-known risk factor for stroke; however, its underlying molecular mechanism remains unclear. Using both mouse and cell culture models, we have provided evidence that impairment of autophagy has a central role in HHcy-induced cellular injury in the mouse brain. We observed accumulation of LC3B-II and p62 that was associated with increased MTOR signaling in human and mouse primary astrocyte cell cultures as well as a diet-induced mouse model of HHcy, HHcy decreased lysosomal membrane protein LAMP2, vacuolar ATPase (ATP6V0A2), and protease cathepsin D, suggesting that lysosomal dysfunction also contributed to the autophagic defect. Moreover, HHcy increased unfolded protein response. Interestingly, Vitamin B supplementation restored autophagic flux, alleviated ER stress, and reversed lysosomal dysfunction due to HHCy. Furthermore, the autophagy inducer, rapamycin was able to relieve ER stress and reverse lysosomal dysfunction caused by HHcy *in vitro*. Inhibition of autophagy by HHcy exacerbated cellular injury during oxygen and glucose deprivation and reperfusion (OGD/R), and oxidative stress. These effects were prevented by Vitamin B co-treatment, suggesting that it may be helpful in relieving detrimental effects of HHcy in ischemia/reperfusion or oxidative stress. Collectively, these findings show that Vitamin B therapy can reverse defects in cellular autophagy and ER stress due to HHcy; and thus may be a potential treatment to reduce ischemic damage caused by stroke in patients with HHcy.

Hyperhomocysteinemia (HHcy) is a clinical condition characterized by increased levels of total plasma homocysteine (Hcy) and carries an increased risk for stroke.^1^ Hcy is a methionine precursor and a sulphur amino acid intermediate in the methylation and trans-sulfuration pathways. There are three major dietary cofactors in Hcy metabolism: Vitamin B_6_, B_12_, and folate. Deficiencies in these vitamins were more prevalent in the developing countries and may account for the increased incidence of HHcy and stroke found in those countries.^2^ Additionally, decreased folate, Vitamin B_6_, and Vitamin B_12_ plasma levels were associated with HHcy;^3^ moreover, Vitamin B therapy reduced both Hcy levels and stroke risk.^4, 5^ HHcy is frequently categorized into three categories: moderate (plasma Hcy concentrations of 15–30 *μ*mol/l), intermediate (plasma Hcy concentrations of 31–100 *μ*mol/l), and severe (plasma Hcy concentrations 100 *μ*mol/l).^6^

Autophagy degrades nonfunctional misfolded proteins in endoplasmic reticulum (ER) to reduce ER stress and counteract the unfolded protein response.^7, 8^ PERK, IRE1, and increased cytosolic calcium are potential mediators of ER stress-induced autophagy in mammalian cells.^9^ Autophagy decreased ER-mediated apoptosis and cell death, and thus may have had a cyto-protective effect against ER stress and nfolded protein response.^10^ In this regard, HHcy has been shown to induce ER stress in hepatocytes, endothelial cells, and vascular smooth muscle,^11^ but its effects on autophagy and the brain have not been studied previously.

The relationship(s) between ER stress and autophagy in HHcy is not well understood. HHcy increased the risk for stroke and caused endothelial cell dysfunction, while Vitamin B supplementation can ameliorate these effects in HHcy.^12^ However, it was not known whether Vitamin B therapy stimulated autophagy and/or decreased ER stress in HHcy. Recent reports have suggested that autophagy may confer neuroprotection in ischemic brain.^13, 14^ Thus, better understanding of these processes could lead to new strategies for neuro-prevention and restoration, reduction of stroke damage, and prevention of stroke. In this study, we used mouse brain and primary human/mouse astrocytes models to show that HHcy inhibited autophagy and was associated with increased MTOR-lysosomal signaling. HHcy also increased nfolded protein response and ER stress. Interestingly, Vitamin B supplementation of the diet or culture medium after HHcy treatment decreased MTOR signaling, which in turn restored autophagy and reactivated the lysosomal pathway. The MTOR inhibitor, rapamycin, mimicked the reversal of Hcy-induced inhibition of autophagy and ER stress. It also counter-acted HHcy-exacerbated damage caused by reactive oxygen species, glucose deprivation and reperfusion (OGD/R) in primary astrocyte cultures. Our findings suggest that Vitamin B supplementation may provide neuroprotection in HHcy, and thereby reduce the risk and extent of stroke in patients with this condition.

## Results

### Hcy treatment impaired autophagy in primary astrocytes

**Figure 1:**
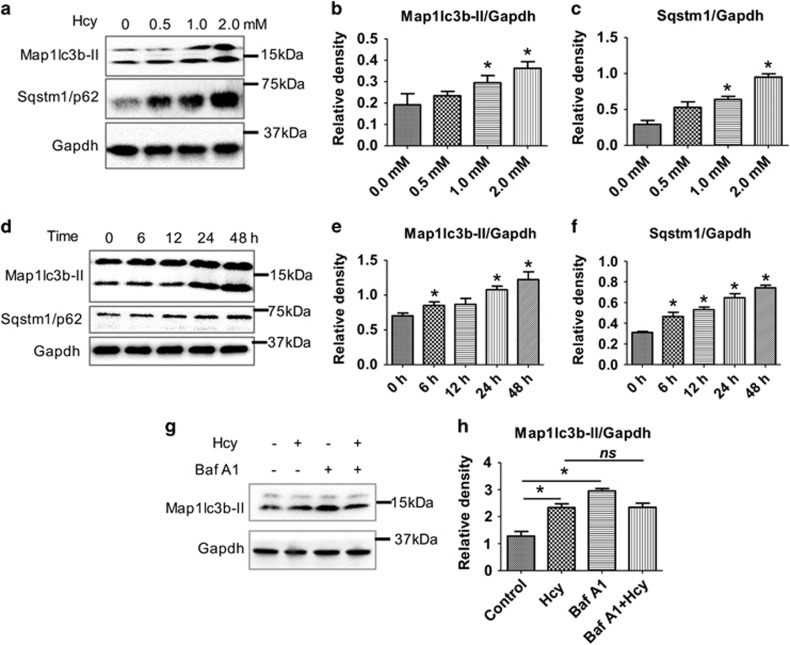
Hcy inhibited autophagy in primary mouse astrocytes. Primary astrocytes were isolated and cultured as described in materials and methods. (**a**) western blot analysis of primary mouse astrocytes treated with different Hcy concentrations as mentioned in the figure panel for 48 h. (**b** and **c**) Quantitative analysis of MAP1LC3B-II and SQSTM1, respectively, was done and plotted as bar graphs. (**d**) Western blot analysis of primary mouse astrocytes treated with 2.0 mM Hcy for different time points as mentioned in the figure panel. (**e** and **f**) Quantitative analysis of MAP1LC3B-II and SQSTM1, respectively was done and plotted as bar graphs. (**g**) Autophagic flux analysis was done by using Bafilomycine A1 (50 nM for 6 h) along with or without Hcy (2.0 mM for 48 h). (**h**) Quantitative analysis of MAP1LC3B-II was done and plotted as bar graphs. The data are shown as Mean±S.D. and statistical difference *P<0.05 was considered as significant

Autophagy is a key mechanism for neuronal survival and functional recovery after stroke.^15^ To examine the effect of HHcy on autophagy, we treated primary mouse astrocytes with varying concentrations of Hcy (0–2 mM) and at different time points. Western blot results revealed that HHcy caused significant increases in the levels of autophagy protein LC3B-II (MAP1LC3B-II) and its adaptor protein SQSTM1/p62 in a dose- (Figures 1a–c) and time- (Figures 1d–f) dependent manner. Next, we performed autophagic flux analysis using the lysosomal inhibitor BafA1^16^ and observed a decrease in autophagic flux by Hcy compared with control samples (Figures 1g and h). Hcy treatment also increased LC3B-II and SQSTM1/p62 levels in a dose-dependent manner in SH-SY5Y human neuroblastoma cells (Supplementary Figure 1A–C).

### Autophagic flux inhibited by Hcy due to increased MTOR signaling and lysosomal dysfunction

**Figure 2:**
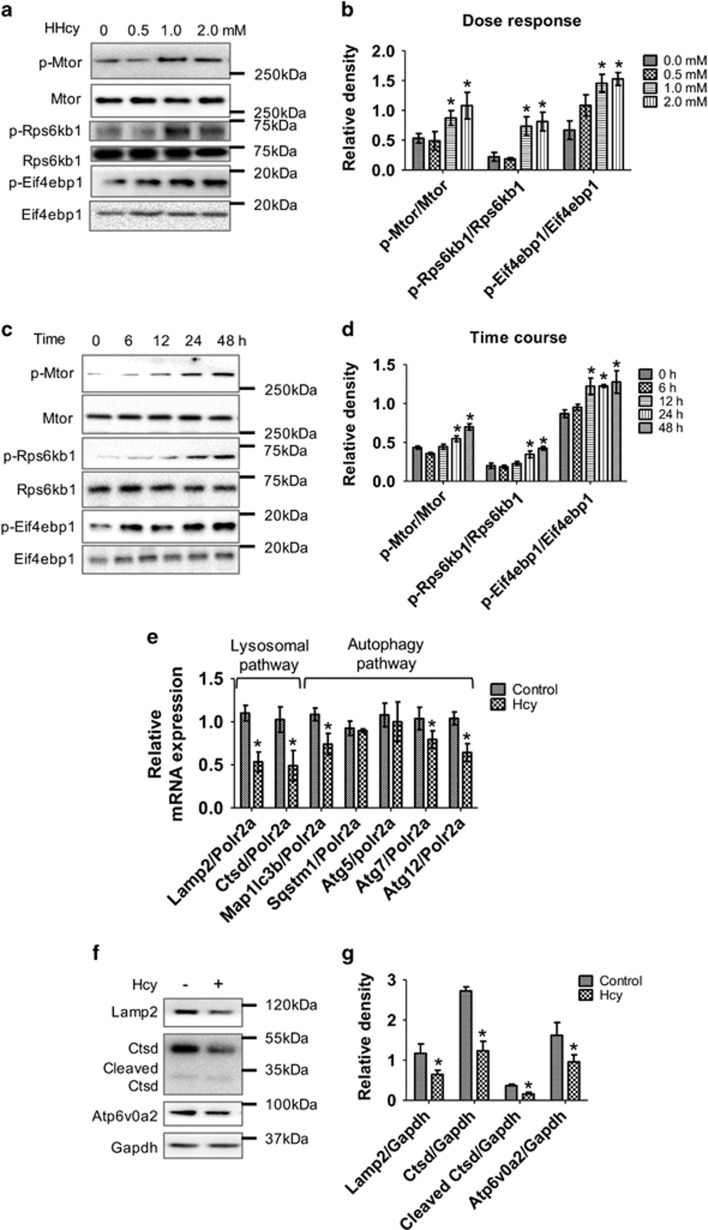
Hcy increased MTOR signaling and inhibited lysosomal pathway in primary mouse astrocytes. Primary astrocytes were isolated and cultured as described in materials and methods. (**a**) Western blot analysis of primary mouse astrocytes treated with different Hcy concentrations as mentioned in the figure panel for 48 h. (**b**) Quantitative analysis of phospho-MTOR, RPS6KB1 and EIF4EBP1 proteins was done and plotted as bar graphs. (**c**) Western blot analysis of primary mouse astrocytes treated with 2.0 mM Hcy for different time points as mentioned in the figure panel. (**d**) Quantitative analysis of phospho-MTOR, RPS6KB1 and EIF4EBP1 proteins was done and plotted as bar graphs. (**e**) Primary mouse astrocytes treated with Hcy (2.0 mM for 48 h), RT-qPCR analysis of key lysosomal and autophagic genes was performed and plotted as bar graph. (**f**) Western blot analysis of primary mouse astrocytes treated with Hcy (2.0 mM for 48 h). (**g**) Quantitative analysis of western blots in **f** was done and plotted as bar graphs. The data are shown as Mean±S.D. and statistical difference *P<0.05 was considered as significant

We observed significant dose- and time-dependent increases in the phosphorylation of MTOR and its downstream substrates RPS6KB1/p70S6K and EIF4EBP1 (Figures 2a–d) in primary astrocytes. Significant increases in levels of MTOR phosphorylation downstream substrates RPS6KB1/p70S6K and EIF4EBP1 could be measured after 24 h treatment (Figures 2c and d). Similarly, Hcy treatment caused significant increases in the level of MTOR phosphorylation and its downstream substrates RPS6KB1/p70S6K and EIF4EBP1 in human neuroblastoma SH-SY5Y cells (Supplementary Figures 2A and B).

Transcription factor EB (TFEB) regulates the expression of lysosomal and autophagic target genes. MTOR can decrease lysosomal function by phosphorylating TFEB to cause cytoplasmic retention and reduce lysosomal gene expression.^17^ We observed that Hcy treatment increased TFEB cytoplasmic retention (Supplementary Figure 2C) and downregulated the expression of lysosomal and autophagy-related target genes under the control of TFEB (Figure 2e). In particular, Hcy treatment decreased *LAMP2* and cathepsin D (*CTSD*) as well as *LC3B, ATG7* and *ATG12* gene expression. Furthermore, lysosomal function was impaired by Hcy, as protein expression of the membrane-associated lysosomal protein 2 (LAMP2), vacuolar ATPase (ATP6V0A2), and CTSD were significantly downregulated (Figures 2f and g). These results suggested that Hcy impaired autophagic flux by increasing MTOR signaling and reducing lysosome gene expression.

### Hcy caused endoplasmic reticulum (ER) stress

**Figure 3:**
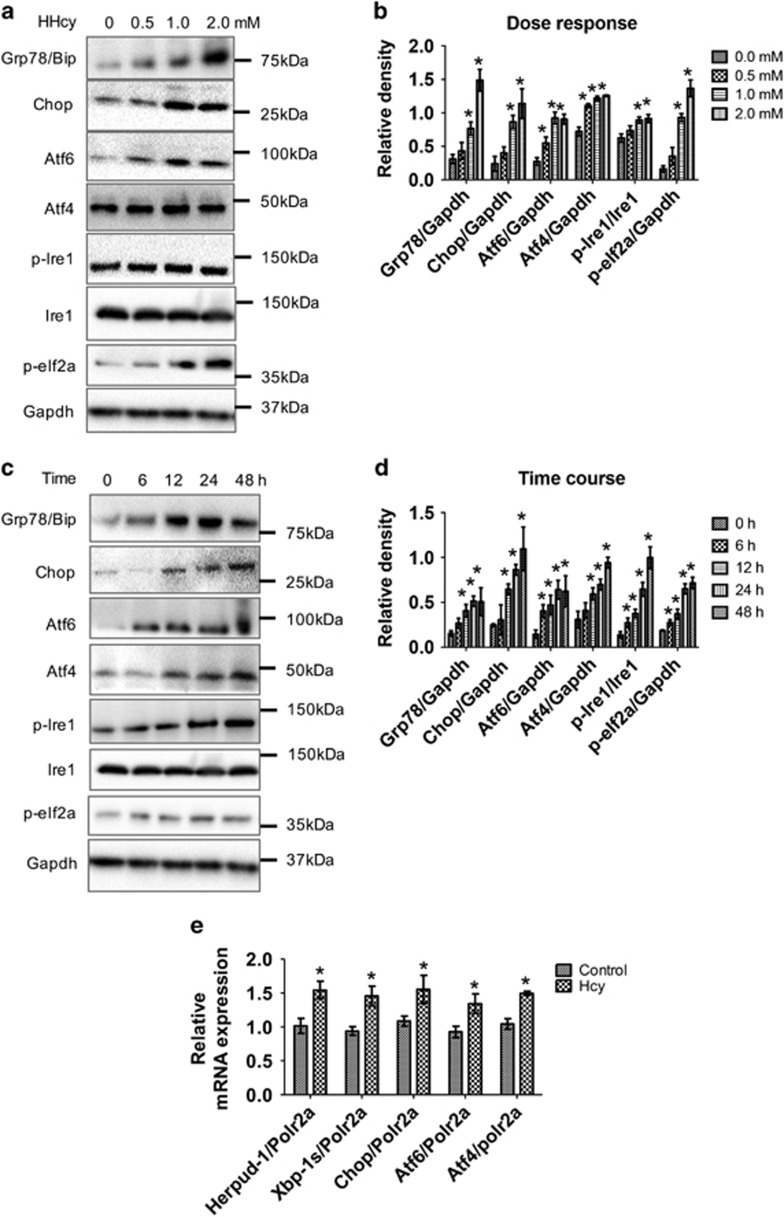
Hcy increased ER stress in primary mouse astrocytes. Primary astrocytes were isolated and cultured as described in materials and methods. (**a**) Western blot analysis of primary mouse astrocytes treated with different Hcy concentrations as mentioned in the figure panel for 48 h. (**b**) Quantitative analysis of ER stress marker proteins was done and plotted as bar graphs. (**c**) Western blot analysis of primary mouse astrocytes treated with 2.0 mM Hcy for different time points as mentioned in the figure panel. (**d**) Quantitative analysis of ER stress marker proteins was done and plotted as bar graphs. (**e**) Primary mouse astrocytes treated with Hcy (2.0 mM for 48 h), RT-qPCR analysis of key ER stress genes was performed and plotted as bar graph. The data are shown as Mean±S.D. and statistical difference *P<0.05 was considered as significant

Since autophagy is an essential protective mechanism during ER stress,^18^ we investigated the effect of Hcy on ER stress. We observed that Hcy caused dose- (Figures 3a and b) and time- (Figures 3c and d) dependent increases in the levels of ER stress marker proteins in mouse primary astrocytes. One of the most characterized ER chaperones is the 78 kDa glucose-regulated protein (GRP78), also referred to as BiP. Hcy increased GRP78 in a dose- and time-dependent manner. We also observed significant induction of the ER stress-associated proapoptotic marker CHOP, and phosphorylation of EIF2a and inositol-requiring enzyme-1 (IRE1). Furthermore, important ER stress response transducers, activating transcription factor 6 (ATF6), and activating transcription factor 4 (ATF4) were upregulated by Hcy treatment in primary mouse astrocytes. We performed similar studies in the SH-SY5Y human neuroblastoma cells, and observed a similar dose-dependent increase in ER stress marker levels (Supplementary Figure 3). Hcy also increased HERPUD-1 XBP-1s, CHOP, ATF6, and ATF4 mRNA expression (Figure 3e). These data suggested that inhibition of autophagy by Hcy was associated with increased ER stress.

### Vitamin B_12_ and folate co-treatment with Hcy induced autophagy and alleviated lysosomal dysfunction and ER stress

**Figure 4:**
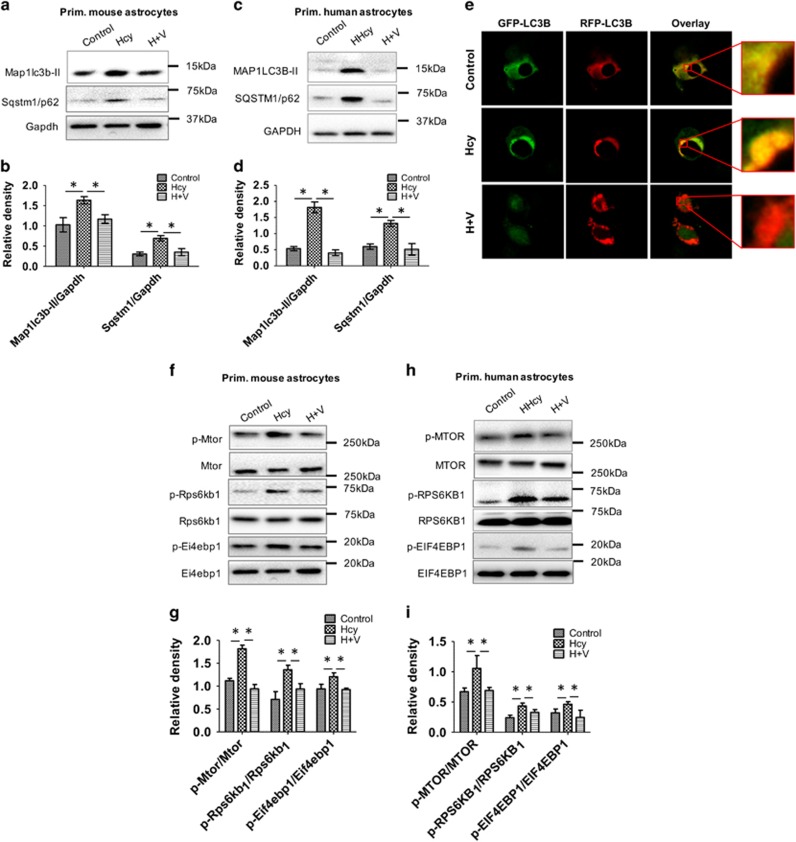
Vitamin B_12_ and folate prevented Hcy-induced autophagic inhibition. (**a** and **c**) Western blot analysis of primary mouse and human astrocytes, respectively treated with Hcy (2.0 mM for 48 h) alone or along with Vitamin B_12_ and folate (1 : 1; 2 mM). (**b** and **d**) Quantitative analysis of MAP1LC3B-II and SQSTM1 proteins was done and plotted as bar graphs. The data are shown as Mean±S.D. and statistical difference *P<0.05 was considered as significant. (**e**) SH-SY5Y neuroclastoma cells were transfected with ptf-LC3B and treated with Hcy (2.0 mM for 48 h) alone or along with Vitamin B_12_ and folate (1 : 1; 2 mM). autophagic flux analysis was then performed by analyzing red puncta formation under confocal microscope at 40 × magnification. Representative micrographs are shown here and enlarged view in the inset. (**f** and **h**) Western blot analysis of primary mouse and human astrocytes, respectively, treated with Hcy (2.0 mM for 48 h) alone or along with Vitamin B_12_ and folate (1 : 1; 2 mM). (**g** and **i**) Quantitative analysis of key MTOR signaling proteins was done and plotted as bar graphs. The data are shown as Mean±S.D. and statistical difference *P<0.05 was considered as significant

Modest Vitamin B supplementation was effective in reducing elevated plasma Hcy concentrations.^19^ Therefore, we next examined the effects of vitamin supplementation (Vitamin B_12_+folate) *in vitro* during Hcy treatment. Primary mouse and human astrocytes as well as neuroblastoma SH-SY5Y cells, were co-treated with Hcy (2 mM) as well as vitamins B_12_, and folate (5 *μ*M; 1 : 1) for 48 h. Protein levels of LC3B-II and SQSTM1/p62 were significantly decreased in the co-treated cells compared with Hcy-treated cells in both primary mouse (Figures 4a and b) and human astrocytes (Figures 4c and d). Furthermore, we confirmed autophagic flux by ectopically expressing tandem fluorescence-LC3B (tf-LC3B) in SH-SY5Y cells treated with Hcy with or without Vitamin B_12_ and folate (Figure 4e). In this assay, RFP tagged to LC3B detected both autophagosomes and autolysosomes, whereas eGFP tagged to LC3B detected only autophagosomes (since eGFP is quenched in acidic pH compartments). Thus, yellow fluorescence occurred in autophagosomes (in overlay image) whereas red fluorescent puncta were found in autolysosomes. This functional assay for autophagy detected autophagosomes relative to autolysosomes (remaining red puncta), in merged images, and was a useful method for characterizing autophagic flux.^20^ Using this technique, we observed that Hcy treatment increased yellow puncta, suggesting accumulation of autophagosomes, whereas Vitamin B_12_ and folate co-treatment with Hcy increased autolysosome (red puncta) formation in merged images, indicating increased autolysosome formation (Figure 4e). Moreover, we observed that cells co-treated with Vitamin B_12_ and folate together with Hcy reduced phosphorylation of MTOR and its downstream targets RPS6KB1/p70S6K and EIF4EBP1 compared with cells treated with Hcy alone in primary mouse (Figures 4f and h) and human (Figures 4g and i) astrocytes.

**Figure 5:**
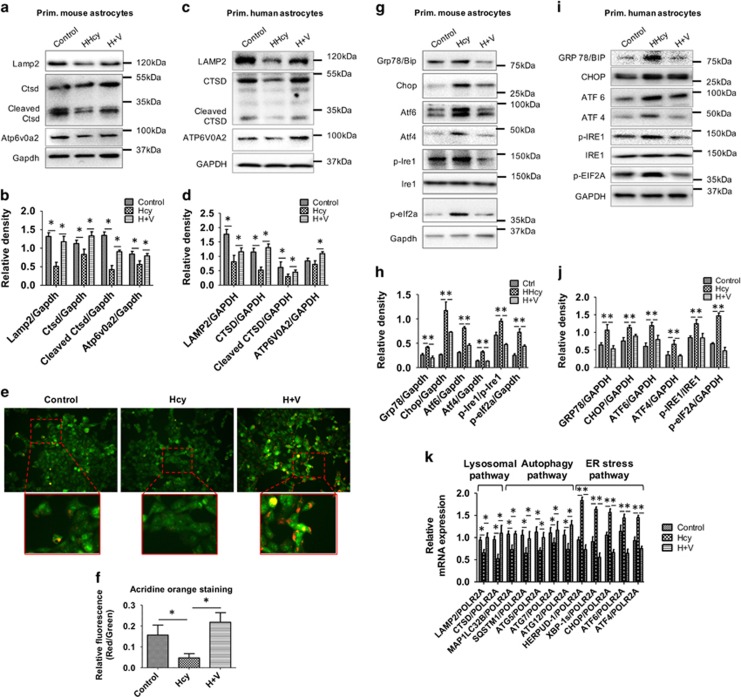
Vitamin B_12_ and folate prevented Hcy-induced decrease in lysosomal pathway. (**a** and **c**) Western blot analysis of primary mouse and human astrocytes, respectively, treated with Hcy (2.0 mM for 48 h) alone or along with Vitamin B_12_ and folate (1 : 1; 2 mM). (**b** and **d**) Quantitative analysis of key lysosomal proteins was done and plotted as bar graphs. (**e**) SH-SY5Y neuroclastoma cells were treated with Hcy (2.0 mM for 48 h) alone or along with Vitamin B_12_ and folate (1 : 1; 2 mM); and stained with AO for 30 min. Acidic compartments (lysosomal vesicles as RED) and nucleus (as GREEN) were observed under fluorescence microscope and captured using LAS software, analyzing red puncta formation under confocal microscope at 20 × magnification. Representative micrographs are shown here and enlarged view in the inset. (**f**) Relative fluorescence of lysosomal vesicles (RED) and nucleus (GREEN) was measured using ImageJ software (NIH, USA) and plotted as bar graph. The data are shown as Mean±S.D. and statistical difference *P<0.05 was considered as significant. (**g** and **i**) Western blot analysis of primary mouse and human astrocytes, respectively, treated with Hcy (2.0 mM for 48 h) alone or along with Vitamin B_12_ and folate (1 : 1; 2 mM). (**h** and **j**) Quantitative analysis of key lysosomal proteins was done and plotted as bar graphs. (**k**) Primary human astrocytes treated with Hcy (2.0 mM for 48 h) alone or along with Vitamin B_12_ and folate (1 : 1; 2 mM), RT-qPCR analysis of key lysosmal, autophagy and ER stress pathway genes was performed and plotted as bar graph. The data are shown as Mean±S.D. and statistical difference *P<0.05 was considered as significant

We also observed that Vitamin B_12_ and folate supplementation contributed to the recovery of lysosomal function. In particular, Vitamin B_12_ and folate co-treatment with Hcy restored LAMP2, vacuolar ATPase (ATP6V0A2), and lysosomal hydrolase CTSD expression in primary mouse (Figures 5a and b) and human (Figures 5c and d) astrocytes. Vacuolar ATPase is an ATP-driven proton pump importing protons into the lysosomal lumen, and is responsible for the acidification of the lysosome to enable the maturation and activation of lysosomal enzymes such as CTSD. Thus, maintenance of acidity is a hallmark of functionally mature lysosomes. Accordingly, we assessed lysosomal acidification using acridine orange (AO) staining. SH-SY5Y cells that were co-treated with Vitamin B_12_, folate, and Hcy showed a dramatic increase in orange/red fluorescence compared with Hcy-treated and control cells (Figures 5e and f), confirming an increase in the number of functioning acidic compartments that is, lysosomes, in cells co-treated with these vitamins.

To directly evaluate the effects of Vitamin B_12_ and folate supplementation on Hcy-induced ER stress and autophagic flux, we performed western blot analysis of ER stress markers GRP78, CHOP, ATF6, and ATF4. Results revealed that ER stress markers were upregulated in Hcy-treated cells and reversed by Vitamin B_12_ and folate co-treatment (H+V) in primary mouse (Figures 5g and h) and human astrocytes (Figures 5i and j), respectively. These findings demonstrated a protective role for Vitamin B_12_ and folate against Hcy-induced ER stress.

The CLEAR (Coordinated Lysosomal Expression and Regulation) gene network regulated by TFEB is a critical control point for lysosomal biogenesis and its function. Previously, it was shown that TFEB regulates the transcription of target genes closely related to lysosomal structure and function, including hydrolases, lysosomal membrane proteins, and the vacuolar ATPase complex.^21^ We thus performed RT-qPCR analysis of lysosome (*LAMP2* and *CTSD*) and autophagy (*MAP1LC3B*, *SQSTM1*/*p62*, *ATG5*, *ATG7*, and *ATG12*) genes (Figure 5k) in primary human astrocytes. Hcy treatment decreased mRNA expression of these genes, whereas Vitamin B_12_ and folate treatment restored their mRNA expression. Furthermore, Vitamin B_12_ and folate co-treatment with Hcy restored mRNA expression of Hcy-responsive endoplasmic reticulum-resident ubiquitin-like domain member 2 protein (*HERPUD-1*) and the ER stress genes, *XBP-1s*, *CHOP*, *ATF6* and *ATF4* (Figure 5k). Collectively, our results strongly suggested that Vitamin B_12_ and folate co-treatment with Hcy was able to prevent lysosomal dysfunction, impairment in autophagic flux, and associated ER stress.

### Increased ER stress correlated with the accumulation of SQSTM1/p62 and MAP1LC3B-II *in vivo* using a diet-induced mouse model of HHcy

To examine the effects of Vitamin B (B_6_, B_12_, and folate) therapy during HHcy *in vivo*, we used a dietary approach to induce HHcy in mice (as described in the Material and Methods) that were divided into three groups: control, methionine without Vitamin B (HHcy) (M^+^B^−^), and methionine with Vitamin B (M^+^B^+^). Mice fed with M^+^B^−^ diet showed a significant increase in Hcy levels compared with controls (plasma total Hcy=80.7±19.93 *μ*mol/l *versus* 7.9±1.4 *μ*mol/l). The M^+^B^+^ diet reversed HHcy in these mice as Hcy levels returned to control levels (plasma total Hcy=8.9±1.1 *μ*mol/l *versus* 7.9±1.4 *μ*mol/l).

**Figure 6:**
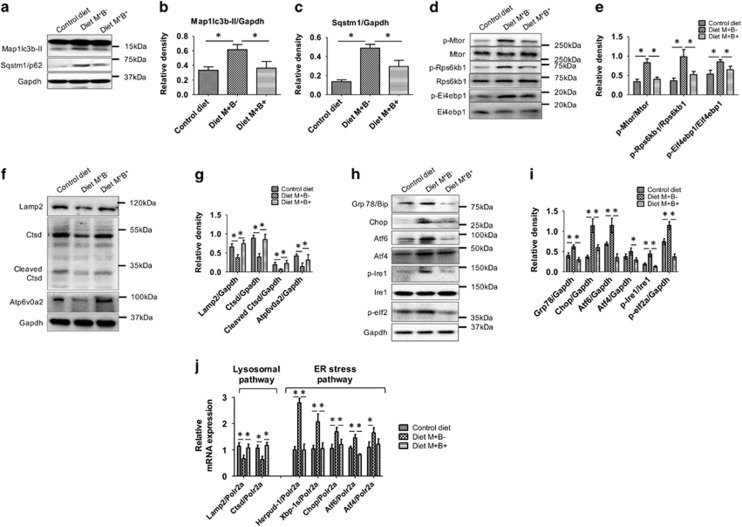
Vitamin supplementation during diet-induced HHcy reversed autophagic block, MTOR signaling activation lysosomal dysfunction and alleviated ER stress in mouse brain. (**a**, **d**, **f,** and **h**) Western blot analysis of brain tissue isolated from mouse fed on control diet, diet rich in Methionine and deficient in vitamin supplementation (M^+^B^−^) caused HHcy; and diet rich in methionine as well as 3 × supplemented with vitamin (M^+^B^+^). (**b** and **c**) Quantitative analysis of MAP1LC3B-II and SQSTM1, respectively, was done and plotted as bar graphs. (**e**, **g,** and **i**) Quantitative analysis of western blots in **d** (for MTOR signaling), **f** (for lysosomal pathway), and **i** (for ER Stress) are plotted as bar graphs. The data are shown as Mean±S.E.M. and statistical difference *P<0.05 was considered as significant. (**j**) RT-qPCR analysis of mouse brain tissues to analyze key genes of lysosomal pathway and ER stress. The data are shown as Mean±S.E.M. and statistical difference *P<0.05 was considered as significant

Western blots of brain tissues from these groups showed that there was increased accumulation of LC3B-II and SQSTM1/p62 proteins from brain tissue in mice fed M^+^B^−^ (HHcy), confirming the autophagic inhibition observed in the cell culture studies (Figures 6a–c). Furthermore, we observed that Diet M^+^B^−^ caused an increase in MTOR phosphorylation and its downstream targets RPS6KB1/p70S6K and EIF4EBP1 (Figures 6d and e). In contrast, there were significant decreases in the levels of LC3B-II and SQSTM1/p62 in brain tissue from mice fed with B_12_, B_6_ and folate supplemented diet (M^+^B^+^), confirming the induction of autophagy in these samples. In the brain tissue of mice fed M^+^B^+^. the expression of the phosphorylated MTOR, RPS6KB1, and EIF4EBP1 was decreased. Mice fed M^+^B^−^ had decreased brain expression of the lysosomal proteins, LAMP2, CTSD, and ATP6V0A2 that was restored to basal levels in the mice fed M^+^B^+^ (Figures 6f and g). Of note, there were no significant changes in expression of autophagy genes *in vivo* (Supplementary Figure 4).

We next examined the expression of ER stress proteins in the same mouse brain tissues. Diet M^+^B^−^ increased expression of ER stress proteins (GRP78, CHOP, ATF6, and ATF4), and the phosphorylation of IRE1 and eIF2a. In contrast, Vitamin B-fortified diet (Diet M^+^B^+^) was able to rescue this increase in ER stress marker proteins (Figures 6h and i). We then examined mRNA expression of genes involved in ER stress (Figure 6j) and found that HHcy increased mRNA levels of *HERPUD-1* and other ER stress markers, *XBP-1 s, ATF6*, *ATF4* and *CHOP* during HHcy treatment that were reversed by Vitamin B co-treatment.

### MTOR inhibitor rapamycin prevented ER stress and induced autophagy in Hcy-treated primary human astrocytes

**Figure 7:**
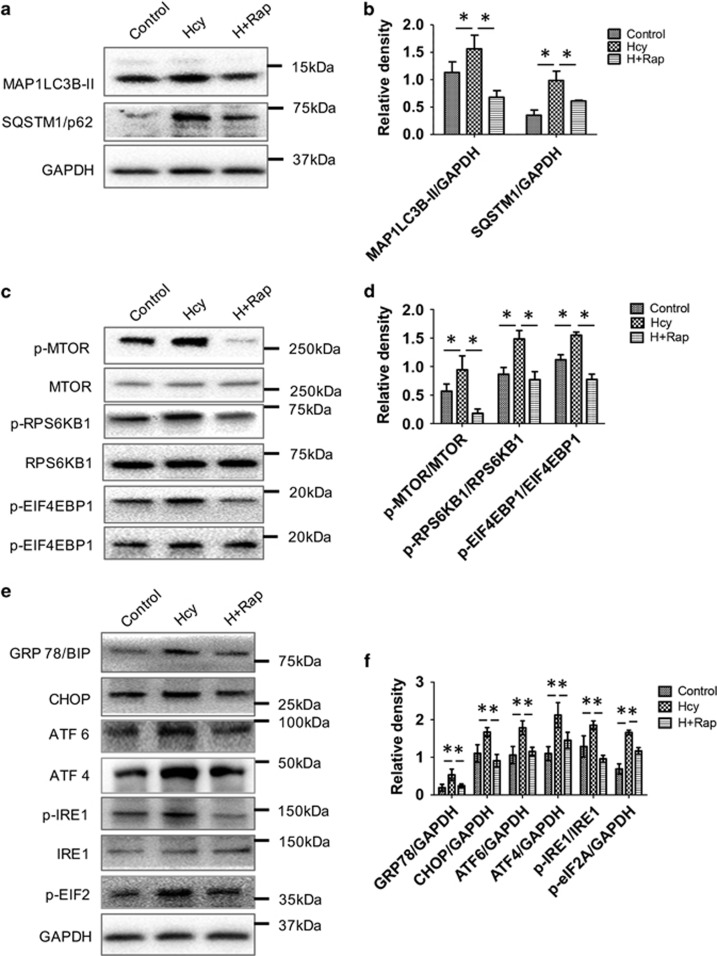
MTOR inhibitor rapamycin treatment reversed Hcy-induced ER stress and inhibition in autophagy. (**a**, **c** and **e**) Western blot analysis of primary human astrocytes treated with Hcy (2.0 mM for 48 h) alone or along with rapamycin (50 *μ*M) for autophagy, MTOR signaling and ER stress, respectively. (**b**, **d** and **f**) Quantitative analysis of western blots was done and plotted as bar graphs. The data are shown as Mean±S.D. and statistical difference *P<0.05 was considered as significant

Our *in vitro* and *in vivo* studies showed that HHcy reduced autophagic flux via stimulation of MTOR signaling and also increased ER stress; moreover, these effects were relieved by Vitamin B therapy. We thus investigated whether a MTOR inhibitor that stimulates autophagy and lysosomal activity such as rapamycin could also relieve HHcy-associated ER stress. Rapamycin co-treatment with Hcy in primary human astrocytes reduced both LC3B-II and SQSTM1/p62 protein levels when compared with Hcy treatment alone (Figures 7a and b), confirming induction of autophagy. We also observed that rapamycin decreased phosphorylation of MTOR and its downstream targets, RPS6KB1/p70S6K and EIF4EBP1 (Figures 7c and d). Additionally, rapamycin co-treatment with Hcy prevented the ER stress induced by Hcy, as evident by significant reductions in the levels ER stress markers GRP78, CHOP, ATF6 and ATF4, and phosphorylation of IRE1 and eIF2a in primary human astrocytes treated with Hcy and rapamycin (Figures 7e and f). These results strongly suggested that autophagic inhibition by HHcy occurred upstream to ER stress and was a cause, rather than a consequence, of increased unfolded protein response.

### Hcy treatment decreased cell survival in an oxygen and glucose deprivation/reperfusion (OGD/R) model and an oxidative stress model

**Figure 8:**
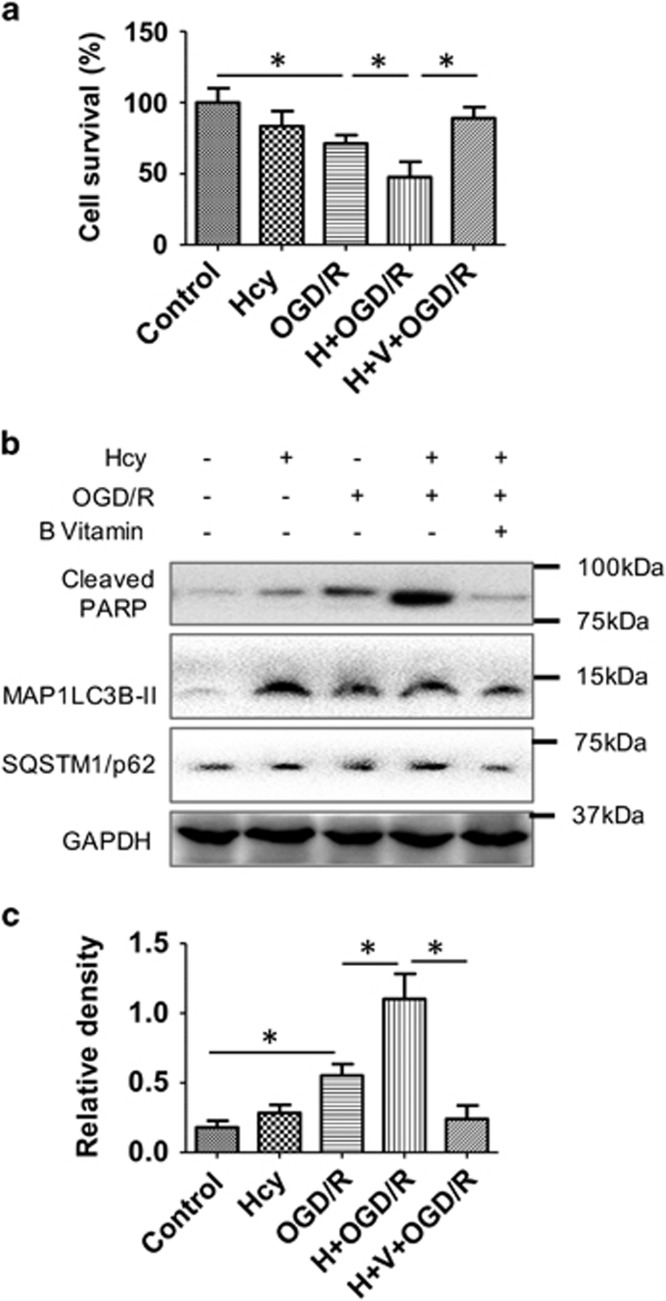
Hcy treatment increased OGD/R-induced damage in primary human astrocytes, which was rescued by vitamin co-treatment. (**a**) Percent cell viability was analyzed by MTS assay as described in material and methods. OGD/R exposure was performed as described in material and methods to mimic I/R *in vitro*. (**b**) Western blot analysis of primary human astrocytes treated with Hcy and Hcy+vitamin, exposed to OGD/R. (**c**) Quantitative analysis of western blots was done and plotted as bar graphs. The data are shown as Mean±S.D. and statistical difference *P<0.05 was considered as significant

From the foregoing *in vitro* and *in vivo* results, we hypothesized that higher levels of Hcy not only had deleterious effects on the cell but also may increase susceptibility to secondary cellular damage after an insult/injury such as ischemia/reperfusion during stroke. Accordingly, we used an OGD/R model to mimic cerebral I/R injury *in vitro*. We observed a 30% decrease in cell survival after OGD/R exposure in primary human astrocytes (Figure 8a), which was further decreased to 46% when cells were pretreated with Hcy prior to ODG/R exposure. In contrast, Vitamin B_12_ and folate supplementation with Hcy increased cell survival to 95% after OGD/R exposure. Furthermore, the cellular apoptotic marker poly-(ADP-ribose) polymerase cleavage (cPARP) also was increased after OGD/R exposure to primary human astrocytes (Figures 8b and c). Hcy pretreatment further enhanced OGD/R-induced cPARP, whereas vitamin supplementation significantly reduced cPARP expression in Hcy+OGD/R-exposed cells. Oxidative stress also can increase cell death during ischemia/reperfusion;^22^ therefore, we used *tert*-BHP, a short-chain organic hydroperoxide that contains a tertiary butyl group and a hydrodioxygen group to induce cell death. This compound produces free radicals that can oxidize organelles nearby that lead to oxidative stress.^23^ *tert*-BHP co-treatment with Hcy in primary human astrocytes decreased cell survival and increased cPARP expression (Supplementary Figures 5A–C). However, vitamin co-treatment with Hcy and *tert*-BHP prevented this decrease in cell survival and decreased cPARP expression. These data strongly suggested that Hcy treatment further exacerbated the deleterious effects of OGD/R and oxidative stress. Vitamin B_12_ and folate supplementation reduced cell death and cPARP expression in Hcy-treated cells that were exposed to both insults.

## Discussion

Hcy is a key metabolic intermediate in sulfur-containing amino acid metabolism. Besides genetic causes of HHcy due to mutations in Methylene tetrahydrofolate reductase (MTHFR), HHcy occurs with deficiencies of folate, B_6_, and B_12_ that normally serve as enzyme co-factors in Hcy metabolism. HHcy is a strong and independent risk factor for vascular diseases, including ischemic cerebral stroke,^24^ and serum levels of Hcy may represent a modifiable risk factor for stroke.^25^ For example, it now is widely recognized that B vitamin therapy reduces Hcy levels and may decrease risk for stroke.^26^ Several studies and meta-analyses, including the HOPE-2 trial, the French SuFolOM3 trial, a subgroup analysis of the Vitamin Intervention for Stroke Prevention Trial that excluded patients with renal failure, and a subgroup analysis of the VITATOPS trial that excluded patients on antiplatelet therapy, all showed a reduced risk of stroke after vitamin supplementation.^27^ Recent studies have suggested that autophagy also promotes survival during stroke.^17^ Stress in the ER compartment of the cell has been implicated in a variety of neural injury models, including ischemic stroke.^28^ Currently, the underlying relationships between ER stress, autophagy, and cell death in the brain during HHcy are not well understood. Moreover, little is known about the interplay of these cellular responses to stressors or injury in the brain.

In this study, we showed that HHcy has deleterious effects on autophagy and ER stress in primary astrocytes and in brain. Our results revealed that higher Hcy level led to accumulation of p62 and LC3B-II proteins consistent with a blockade of autophagy flux (Figures 1a and b; Figures 4a–d; and Figures 6a–c). Moreover, HHcy increased ER stress (Figures 6f and g). Under physiological conditions, induction of autophagy serves as a protective mechanism in response to ER stress. However, our data showed that inhibition of autophagic flux during HHcy significantly increased expression of ER stress markers such as CHOP, XBP-1s, ATF6, ATF4, and GRP78/BIP (Figures 3e and 6j). Of note, GRP78 previously was shown to interact directly with apoptotic pathway intermediates, block caspase activation, and inhibit apoptosis and cell death.^29^ Thus, it is likely that autophagic inhibition contributes and/or exacerbates ER stress in the brain. Interestingly, Vitamin B_12_, B_6_, and folate supplementation of the methionine diet for mice with HHcy both induced autophagy and reduced ER stress markers to basal levels (Figures 6a–c).

Elevated serum Hcy levels previously were shown to be associated with impaired autophagy in the brain but the mechanism was not identified.^30^ Here we showed that increased Hcy concentrations *in vitro* and *in vivo* impaired autophagy by stimulating MTOR-lysosomal signaling. MTOR signaling previously was shown to inhibit autophagosome formation.^31^ Although we observed that increased Hcy level upregulated MTOR signaling (Figures 2a–d; Figures 4h–i; and Figures 6d–e), Hcy treatment surprisingly increased both LC3B-II and SQSTM1/p62 expression, suggesting that there also was a late block in autophagy (Figures 1a–c) occurring after autophagosome formation, involving either autophagsome/ lysosome fusion or lysosomal degradation.^32^

MTOR regulates lysosomal biogenesis and activity by phosphorylating TFEB and decreasing its nucleo-cytoplasmic translocation.^33^ Interestingly, we found that Hcy treatment increased TFEB cytoplasmic localization presumably due to TFEB phosphorylation by MTOR (Supplementary Figure 2C). To further understand autophagy inhibition by Hcy, we investigated expression of lysosomal proteins and their functions. Hcy decreased expression of key lysosomal proteins LAMP2, ATP6V0A2, and CTSD expression in mouse brain and in primary astrocytes (Figures 6f and g). There also was decreased lysosomal acidification in astrocytes treated with Hcy (Figures 6e and f). Our data strongly suggested that impairment in lysosomal function led to the accumulation of autophagic proteins, LC3B-II and SQSTM1/p62. Furthermore, the decreased LAMP2 (membrane protein) and CTSD protein levels in HHcy suggested a decrease in the number of lysosomes and/or insufficient autophagosome/lysosomal fusion. The decrease in vacuolar ATPase (ATP6V0A2) expression by Hcy likely reduced lysosomal acidification. Interestingly, Vitamin B supplementation *in vitro* (B_12_ and folate) and *in vivo* (B_6_, B_12_, and folate) significantly reversed HHcy-induced changes in MTOR signaling and lysosome formation/function (Figures 6d–g). Taken together, these findings showed that Hcy inhibited autophagy by upregulating MTOR signaling and inhibiting the lysosomal pathway, and these effects caused by HHcy could be reversed by Vitamin B supplementation.

Rapamycin is a FDA-approved immunosuppressant and MTOR inhibitor that is widely used during organ transplantation. It has been reported to reduce injuries in several models of neurodegenerative disorders by inducing MTOR-dependent autophagy.^34^ Sheng *et al.* also demonstrated neuroprotection properties of rapamycin in rat focal cerebral ischemia preconditioning.^35^ Moreover, it has been reported that rapamycin provided neuroprotection in a neonatal-hypoxia-ischemia model by activating autophagy and inhibiting apoptosis.^36^ Interestingly, we found that rapamycin reversed the autophagic inhibition and ER stress in primary mouse astrocytes-treated HHcy (Figures 7a–f).

Hcy levels are associated with secondary vascular events and mortality after stroke.^27^ We thus examined the effect of Vitamin B supplementation (B_12_ and folate) on cell viability after exposure of stress in cells treated with Hcy. Primary human astrocytes treated with Hcy and preconditioned by OGD/R or oxidative stress had increased cell death (Figures 8a–f). Vitamin B_12_, folate co-treatment with Hcy significantly reduced the harmful effects of OGD/R and oxidative stress, and improved cell survival. These findings suggest that it is possible that Vitamin B therapy may reduce cell death after exposure to a major stressor such as stroke.

Our findings may have clinical implications as therapies aimed to restore the autophagic flux in patients with HHcy, may prevent stroke and or reduce neuronal damage after a stroke. Vitamin B therapy reduced ER stress and cell death in mouse brain and primary astrocytes and neuroblastoma cells during HHcy. Since vitamins restored lysosomal function and autophagy flux during HHcy (Supplementary Figure 6), it is possible that drugs that have effects on these pathways may have similar beneficial effects. Indeed, we found that rapamycin was able to restore autophagy and reduce ER stress in primary astrocytes treated with Hcy. Thus, drugs that enhance autophagy and lysosomal function may be potentially useful for the prevention and treatment(s) for stroke in patients with HHcy.

## Material and Methods

### Drugs and reagents

D,L-Homocysteine, Vitamin B_12_, Folate, Luperox TBH70X (tert-Butyl hydroperoxide solution), Rapamycin and Acridine Orange (AO) were purchased from Sigma-Aldrich (MO, USA). Antibodies were procured from Cell Signaling Technologies (Danvers, MA, USA) (LC3B, 2775; SQSTM1/p62, 5114; MTOR (7C10) rabbit mAb, 2983; phospho-MTOR (Ser2448) (D9C2), p-eIf2alpha, 9722 S; RPS6KB1/p70S6 kinase (49D7) rabbit mAb, 2708; phospho-RPS6KB1/p70S6 kinase (Thr389) (108D2) rabbit mAb, 9234; GAPDH (D16H11); ATF6 (70B1413.1, mouse Ab, Novus Biologicals, Littleton, CO, USA), NBP1-40256; p-IRE1alpha, rabbit Ab, NB100-2323; IRE1alpha rabbit Ab, (14C10), 3294P, Novus Biologicals; ATP6V0A2, ab96803, rabbit Ab, Abcam (Cambridge, MA, USA); CHOP (L63F7) mouse Ab, 2895 S; Cleaved PARP (D214), rabbit Ab, 9544P; LAMP2 (CD107b) rabbit Ab, Thermo Fisher Scientific Inc., CA, USA; Xbp-1s, (M-186), sc-7160, rabbit Ab; Bip-GRP78, (C50B12), rabbit Ab; Anti-CTSD (SAB2106553), rabbit Ab. Culture media and transfection reagents were from Invitrogen, Thermo Fisher Scientific Inc., CA, USA. ptfLC3 (Plasmid No. 21074) was procured from Addgene (Cambridge, MA, USA) and TFEB plasmid (Plasmid No. 38119) was also procured from Addgene, SHY transfection (DNA-In® SY5Y, GST-2151) reagent was procured from MTI-Globalsystem.com (Thermo Fisher Scientific Inc.), S-adenosyl methionine (SAM) (CEG414Ge) kit was procured from Cloud clone Corp (Houston, USA). HHCy diet was customized by Harlan Teklad, Madison, WI, USA.

### Animal maintenance and diet-induced hyperhomocysteinimia mice model

Male C57BL/6 mice (8- to 10-wk-old) were purchased and housed in hanging polycarbonate cages under a 12 h/12 h light/dark schedule. Animals were killed in CO_2_ chambers. All mice were maintained according to the Guide for the Care and Use of Laboratory Animals (NIH publication no. One.0.0. Revised 2011), and experiments were approved by the IACUCs at Singhealth (2013/SHS/848).

Dietary approach was used to generate the HHcy mouse model.^37^ Diet was customized by Harlan Teklad. Mice (*n*=32) were randomized and divided in two groups, group I (*n*=16) received control diet (AIN-93M), whereas group II (*n*=16) received diet rich in Methionine and deficient in vitamin supplementation (M+B−), which induced HHcy. After 12 weeks, 8 mice from each group was killed and Hcy level was measured in plasma. Remaining mice from group II, now considered as group III, were put on diet rich in methionine and 3X supplemented with vitamin (M^+^B^+^) for 12 weeks. Once confirmed as HHcy model, mice were killed and whole brain was taken out and freezed in liquid nitrogen for further total RNA and protein isolation. Their serum Hcy was measured at every 4-week interval for 24 weeks (Supplementary Figure 7). For blood serum Hcy measurement, blood was collected by submandibular bleeding.^38^ We have also measured their s-adenosyl methionine (SAM)/s-adenosyl homocysteine (SAH) ratio (Supplementary Table 1).

All the diets contained 1% sulfathiozole (10 g/kg, Sigma-Aldrich, MO, USA), a nonabsorbed sulpha drug to inhibit folate formation by gut bacteria just to ensure that the animal's only source of available folate is from diet.

### Homocysteine detection

Mouse Homocysteine Kit (Crystal Chem, Chicago, IL, USA Catalog No.80440): Homocysteine was evaluated in mouse serum according to manufacturer's protocol.

### Cell culture and maintenance

#### Primary mouse astrocytes

Mouse pups postnatal 1–3 days were anesthetized and decapitated. The brain is dissected in cold PBS under microscope. After the olfactory bulb and hippocampus removed, the cortex is collected and then trypsinized for 30 min at room temperature. After trypsinization, the cortex is washed with glia cell culture medium (Gibco, Thermo Fisher Scientific Inc.) for 3 times and is then triturated with 2 ml pipette till no chunks are observed. The glia cells are collected by centrifuge 1000 × *g* for 5 min. The supernatant is removed and the cells are suspended and seeded in Poly-D-Lysine coated T75 flask. After 1 week culturing in 37 °C incubator with 5% CO_2_, the T75, the cells get confluent. Then the T75 flask is moved onto an orbital shaker and shaked for 200 r.p.m overnight to remove the microglias and oligodentrocytes. The purity of primary astrocytes were confirmed by GAFP (astrocyte-specific marker) staining. Purified astrocytes were then collected for experiment and maintained in astrocytes culture medium that was DMEM-supplemented with 15% fetal bovine serum, 1% Penicillin/Streptomycin, 2 mM L-glutamine, 0.1 mM nonessential amino acids.

#### Primary human astrocytes

Primary human astrocytes were purchased from Lonza (Clonetics, Lonza, Walkersville, MD, USA) Normal Human Astrocytes (NHA). These cells were cultured and maintained using astrocytes growth medium bullet kit (AGM Bullet kit: CC-3187 & CC-4123) containing astrocytes basal medium, growth factors, cytokines and required supplements, as per manufacturer's description.

#### SH-SY5Y neuroblastoma cell culture

SH-SY5Y, a human-derived neuroblastoma cell line, was procured from ATCC (Manassas, VA, USA) (ATCC CRL-2266). SH-SY5Y cells are often used as *in vitro* models to study diverse neuronal function in neurobiology.^39^ SH-SY5Y cells were cultured with complete medium (DMEM with 10% fetal bovine serum, 1% Penicillin/Streptomycin) and are passaged every 3–4 days.

#### Vitamin treatment

Hcy and Vitamin B_12_ were dissolved in water whereas Folate was dissolved in 0.1 M NaOH. Compounds were diluted in culture medium to final concentration of 2 mM (Hcy) or 5 *μ*M (Vitamin B_12_ and Folate). For dose-dependent study, Hcy was serially diluted in culture medium to obtain 0.5, 1.0, and 2.0 mM.

### Oxygen and glucose deprivation/reperfusion (OGD/R)

To mimic ischemic-reperfusion like conditions *in vitro*, cell cultures were exposed to oxygen-glucose deprivation/reoxygenation (OGD/R).^40^ This existing model could mimic extracellular conditions in cerebral ischemia and subsequently reperfusion, and is commonly used as a model for *in vitro* research.^41^ In brief, to induce OGD/R injury, cultured primary human astrocytes were cultured in 12-well plate. After 90% confluency, rinsed and incubated in a glucose-free Hank's balanced salt solution (HBSS) composed of 140 mM NaCl, 1.2 mM MgSO_4_, 1.7 mM CaCl_2_, 3.5 mM KCl, 10 mM HEPES, 5 mM NaHCO_3_, and 0.4 mM KH_2_PO_4_ (pH 7.3). Then, the cultures were placed into a specialized, humidified chamber filled with 95% N_2_ and 5% CO_2_ at 37 °C for 6 h. Controls were incubated with the HBSS buffer containing 5.6 mM glucose in a humidified incubator with 5% CO_2_ at 37 °C for the same times as the OGD-R cultures. After the challenge, cultures were transferred to normal culture medium and returned to normoxic conditions for 12 h to mimic reperfusion. The indicated concentrations of HHcy and vitamin supplementation was added to the culture medium 12 h before OGD-R and maintained until the end of recovery.

### *In vitro* transfection and confocal microscopy

Human neuroblastoma SH-SY5Y cells grown on poly-D-lysine-coated chambered slides till 90% confluency. ptfLC3 (Plasmid No. 21074) or pEGFP-N1-TFEB (plasmid No. 38119) plasmids were transfected using DNA-In SY5Y (GST-2151; MTI-GlobalStem) as described in manufacturer's protocol. ptfLC3 was a gift from Tamotsu Yoshimori and described elsewhere,^42^ whereas pEGFP-N1-TFEB was a gift from Shawn Ferguson.^43^ After 24 h of transfection, cells were treated with Hcy and/or Vitamin B_12_+folate for further 48 h. The cells were then fixed with 4% paraformaldehyde for 15 min and washed three times with PBS. Slides were washed and wet mounted in VECTASHIELD Antifade Mounting Medium. Fluorescence imaging was performed using LSM710 Carl Zeiss (Carl Zeiss Microscopy GmbH, Oberkochen, Germany) confocal microscope at 40 × magnification.

### Acridine orange (AO) staining

Cells were grown on poly-D-lysine coated 24-well plate and treated with Hcy and/or Vitamin B_12_+Folate for 48 h. Thereafter, cells were incubated with either 1 μg/ml of AO (Sigma-Aldrich) in PBS for 30 min at 37 °C, followed by three washes with PBS, and then immediately observed under a fluorescence microscope.

### MTS assay

MTS Cell Proliferation Colorimetric Assay Kit (K300–500) was procured from Bio Vision (Milpitas, CA, USA) and instructions were followed as manufacturer's protocol to obtain results.

### Western blot analysis

Cells and tissue samples were lysed using CelLytic M Cell Lysis Reagent (Sigma, C2978). Proteins samples were prepared in 2 × Laemmli Sample Buffer (BioRad No. 1610737, Hercules, CA, USA), separated on SDS-PAGE and immunoblotted using standard protocol. Densitometric analysis was performed using ImageJ software (NIH, Bethesda, MD, USA).

### RNA isolation and real-time PCR

Total RNA was isolated using Qiazol (Qiagen, Hilden Germany) and RT-qPCR was performed using the QuantiTect SYBR Green PCR Kit (Qiagen, 204141) in Rotor-Gene Q (Qiagen) according to the manufacturer's instructions. Primer details can be provided upon request.

### Quantitative and statistical analysis

Results were expressed as Mean±S.D. (for all *in vitro* experiments) or S.E.M. (for all *in viv*o experiments). The statistical significance of differences *P<0.05 was assessed by one-way ANOVA followed by Tukey's *post-hoc* test using PRISM (Graph Pad) for comparing the different groups. Student's *t*-test was used for comparing control *versus* treated groups.
