# Supplementary material for: Hyperhomocysteinemia causes ER stress and impaired autophagy that is reversed by Vitamin B supplementation
Source: Cell Death Dis. 2016 Dec 8;7(12):e2513–. doi: 10.1038/cddis.2016.374 (PMC5260994; doi:10.1038/cddis.2016.374)
Supplement: Supplementary Information [file cddis2016374x1.doc]

**Figure legends:**

**Supplementary Figure 1. Hcy inhibited autophagy in SH-SY5Y neuroblastoma.** SH-SY5Y cells were cultured as described in materials and methods. (**A**) Western blot analysis of SH-SY5Y cells treated with different Hcy concentrations as mentioned in the figure panel for 48 h. (**B** and **C**) Quantitative analysis of MAP1LC3B-II and SQSTM1 respectively was done and plotted as bar graphs. Data is shown as Mean±SD and statistical difference **P*<0.05 was considered as significant.

**Supplementary Figure 2. Effect of Hcy on MTOR signaling and TFEB nuclear translocation in SH-SY5Y neuroblastoma cells.** SH-Sy5Y cells were cultured as described in materials and methods. (**A**) Western blot analysis of SH-SY5Y cells treated with different Hcy concentrations as mentioned in the figure panel for 48 h. (**B**) Quantitative analysis of phospho-MTOR, RPS6KB1 and EIF4EBP1 proteins was done and plotted as bar graphs. (**C**) SH-SY5Y neuroblastoma cells were transfected with TFEB plasmid and treated with Hcy (2.0 mM for 48 h) alone or along with vitamin B12 and folate (1:1; 2 mM). Hcy caused decrease in translocation of TFEB from cytoplasm to nucleus. TFEB translocation analysis was then performed by analyzing red puncta formation under confocal microscope at 40x magnification. Data is shown as Mean±SD and statistical difference **P*<0.05 was considered as significant.

**Supplementary Figure 3. Hcy increased ER stress in SH-SY5Y neuroblastoma cells.** SH-SY5Y cells were cultured as described in materials and methods. (**A**) Western blot analysis of SH-SY5Y cells treated with different Hcy concentrations as mentioned in the figure panel for 48 h. (**B**) Quantitative analysis of ER stress marker proteins were done and plotted as bar graphs. Data is shown as Mean±SD and statistical difference **P*<0.05 was considered as significant.

**Supplementary Figure 4. mRNA expression of autophagic genes.** RT-qPCR analysis of mouse brain tissues to analyze key genes involved in autophagic pathway. mRNA was isolated from brain tissue isolated from mouse fed on control diet, diet rich in Methionine and deficient in vitamin supplementation (M^+^B^-^) caused hyperhomocysteinemia; and diet rich in methionine as well as 3X supplemented with vitamin (M^+^B^+^). Data is shown as Mean±SEM and statistical difference **P*<0.05 was considered as significant.

**Supplementary figure 5. Hcy treatment increased *tert*-BHP-induced damage in primary human astrocytes, which was rescued by vitamin co-treatment.** (**A**) Percent cell viability was analyzed by MTS assay as described in material and methods. t-BHP exposure was performed as described in material and methods to induce oxidative stress. (B) Western blot analysis of primary human astrocytes treated with Hcy and Hcy + vitamin, exposed to t-BHP. (C) Quantitative analysis of western blots was done and plotted as bar graphs. Data is shown as Mean±SD and statistical difference **P*<0.05 was considered as significant.

**Supplementary figure 6: Proposed restoration mechanism of vitamin supplementation during Hcy-induced derangement:** Vitamin Supplementation caused restoration of Hcy induced derangement by decreasing MTOR signaling and restoring lysosomal dysfunction which in turn activates autophagy and reduces ER stress.

**Supplementary figure 7. Level of Hcy in serum of diet induced mice model.** Evaluation of Hcy in serum was done according to manufacturer’s protocol. Mouse fed on control diet did not show any changes in Hcy level, diet rich in Methionine and deficient in vitamin supplementation (M^+^B^-^) showed increase in serum Hcy level; and diet rich in methionine as well as 3X supplemented with vitamin (M^+^B^+^) showed decrease in Hcy level. Data is shown as Mean±SD and statistical difference **P*<0.05 was considered as significant.
