# Supplementary figures and images for: Hyperhomocysteinemia causes ER stress and impaired autophagy that is reversed by Vitamin B supplementation
Source: Cell Death Dis. 2016 Dec 8;7(12):e2513–. doi: 10.1038/cddis.2016.374 (PMC5260994; doi:10.1038/cddis.2016.374)

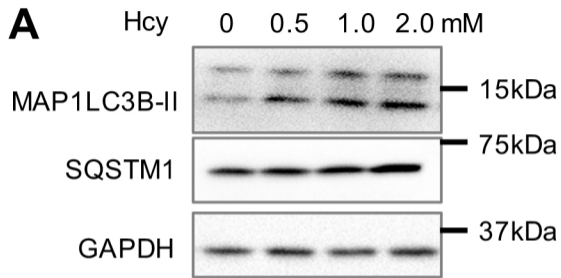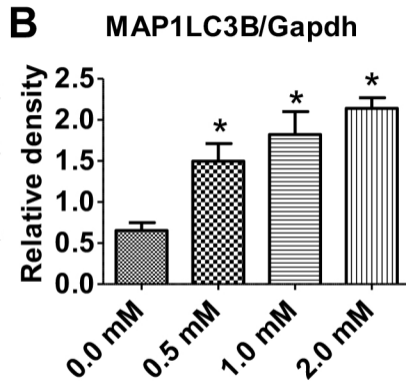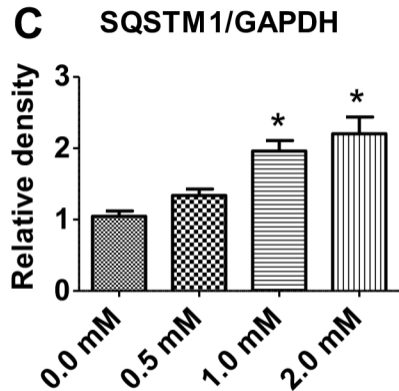

Supplement: Supplementary Figure 1 [file cddis2016374x2.pdf]

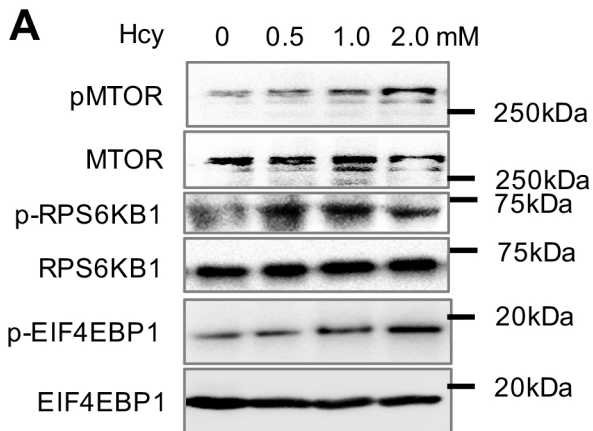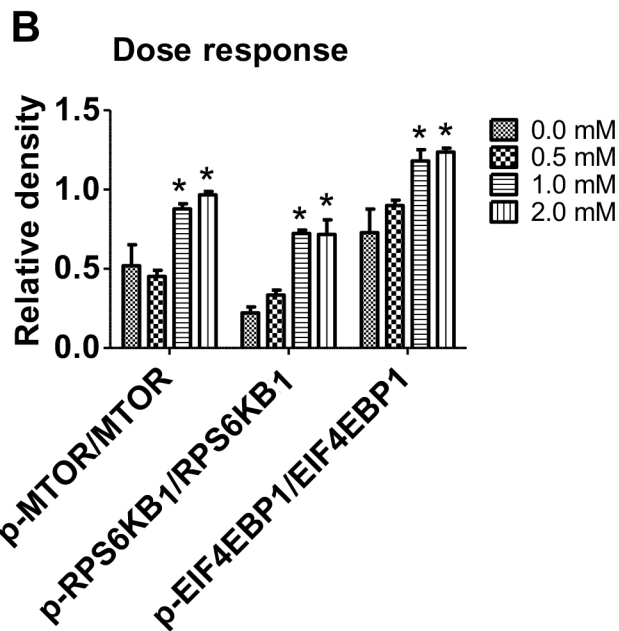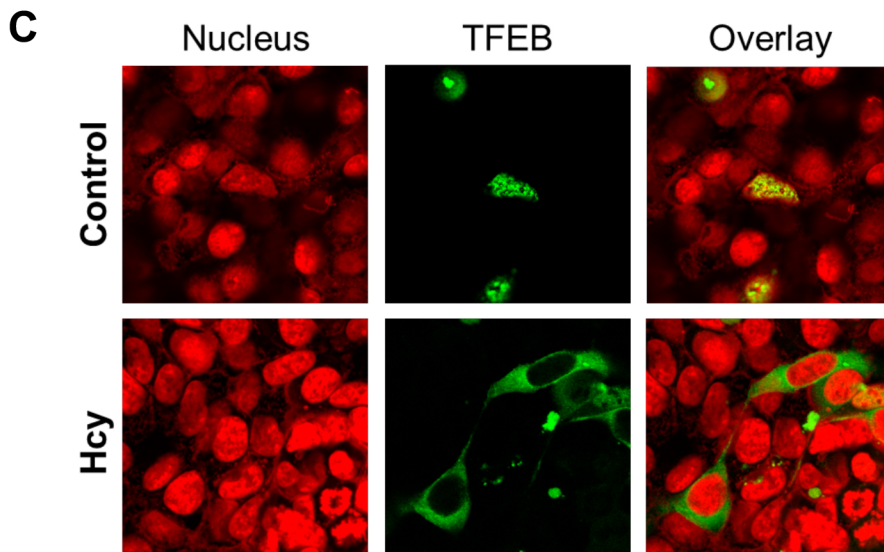

Supplement: Supplementary Figure 2 [file cddis2016374x3.pdf]

**A**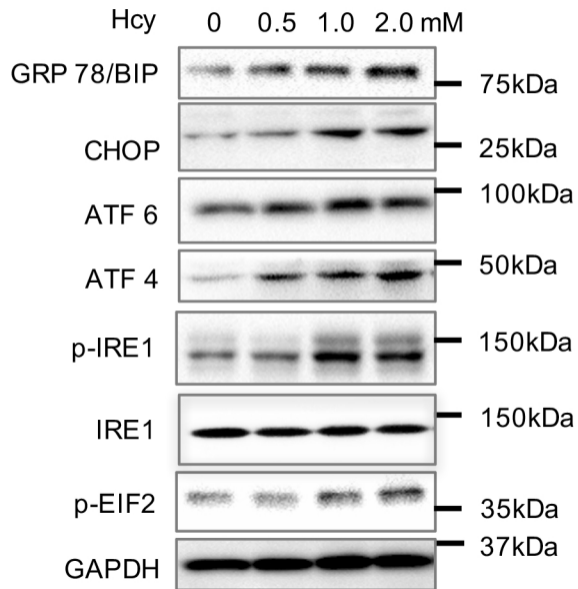**B**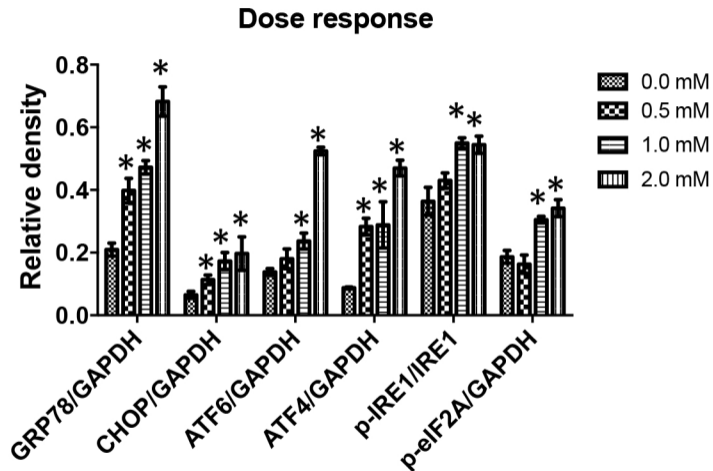

Supplement: Supplementary Figure 3 [file cddis2016374x4.pdf]

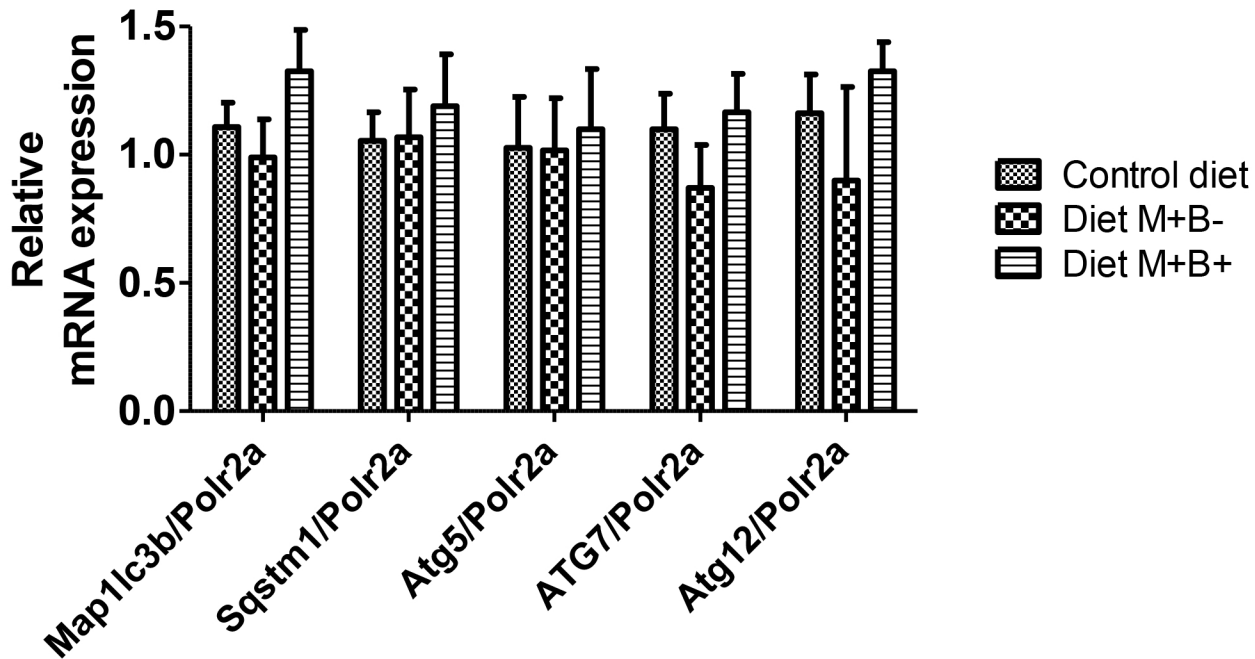

Supplement: Supplementary Figure 4 [file cddis2016374x5.pdf]

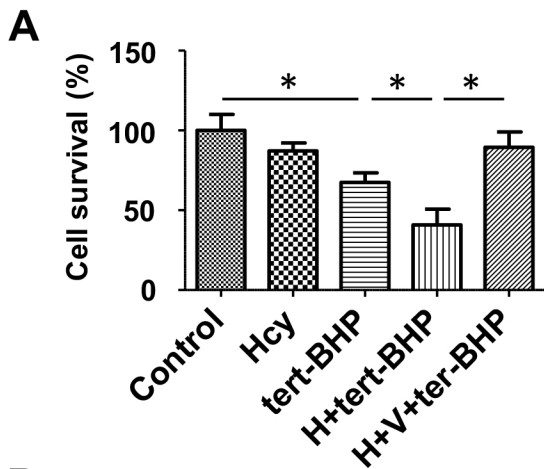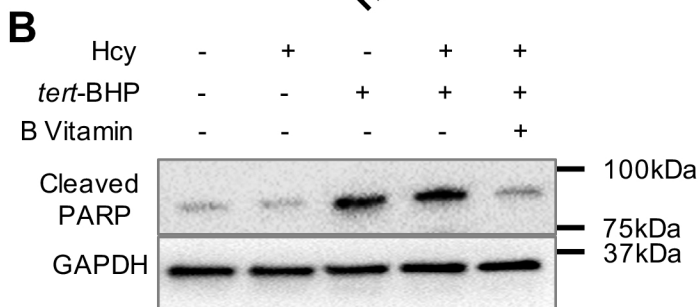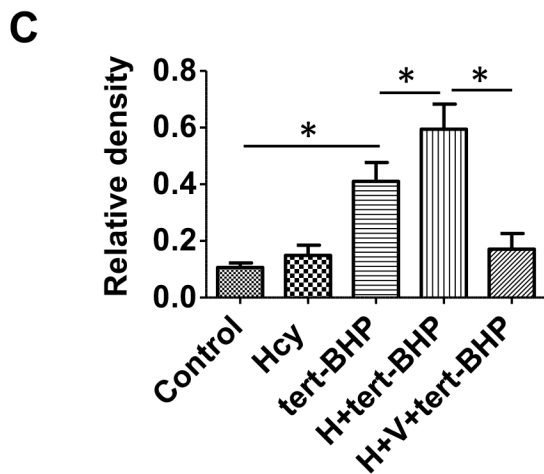

Supplement: Supplementary Figure 5 [file cddis2016374x6.pdf]

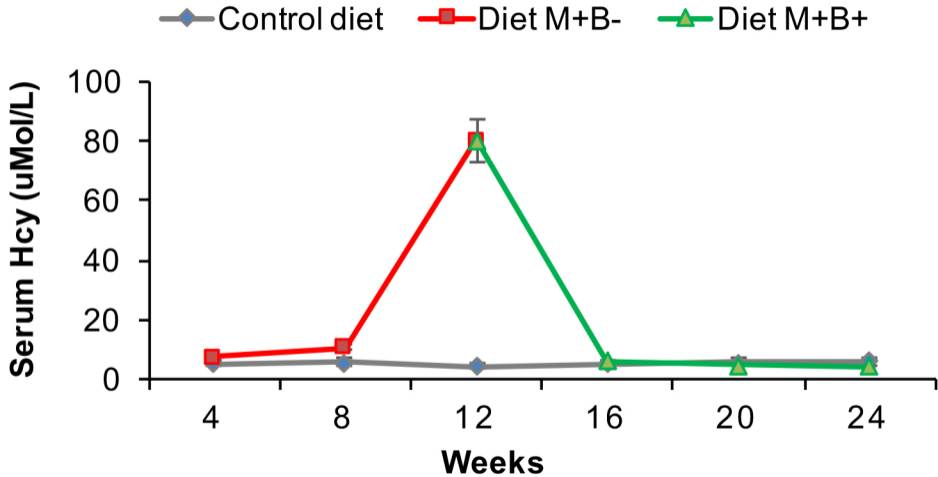

Supplement: Supplementary Figure 7 [file cddis2016374x8.pdf]
