# Supplementary material for: Hyperhomocysteinemia causes ER stress and impaired autophagy that is reversed by Vitamin B supplementation
Source: Cell Death Dis. 2016 Dec 8;7(12):e2513–. doi: 10.1038/cddis.2016.374 (PMC5260994; doi:10.1038/cddis.2016.374)
Supplement: Supplementary Figure 6 [file cddis2016374x7.pdf]

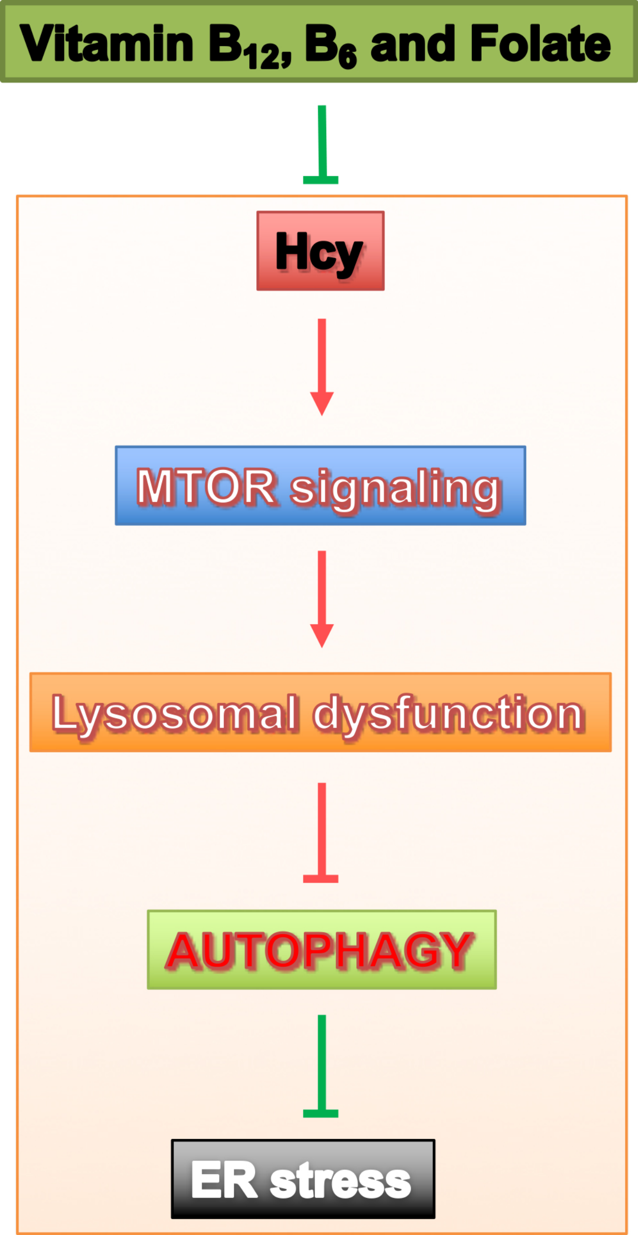
Vitamin B12, B6 and Folate
Hcy
MTOR signaling
Lysosomal dysfunction
AUTOPHAGY
ER stress
