# Supplementary material for: Hyperhomocysteinemia causes ER stress and impaired autophagy that is reversed by Vitamin B supplementation
Source: Cell Death Dis. 2016 Dec 8;7(12):e2513–. doi: 10.1038/cddis.2016.374 (PMC5260994; doi:10.1038/cddis.2016.374)
Supplement: Supplementary Table 1 [file cddis2016374x9.pdf]

Diet	SAM, nmol/g	SAH, nmol/g	SAM/SAH
Control (AIN93M)	12.8±0.5	1.4±0.14	**9.14**
M^+B^- Methionine rich B Vitamin deficient	13.2±0.8	3.2±0.3	**4.125**
M^+B^+ Methionine rich B Vitamin rich	12.0±0.4	1.08±0.2	**11.11**
